# Supplementary material for: A Humanized Yeast Phenomic Model of Deoxycytidine Kinase to Predict Genetic Buffering of Nucleoside Analog Cytotoxicity
Source: Genes (Basel). 2019 Sep 30;10(10):770. doi: 10.3390/genes10100770 (PMC6826991; doi:10.3390/genes10100770)
Supplement: Supplementary file 1 [file genes-10-00770-s001.zip › Additional_Files/Additional_File7_GOTermHeatmaps.zip!/Additional_File7_GOTermHeatmaps/nucleotide_binding.pdf]

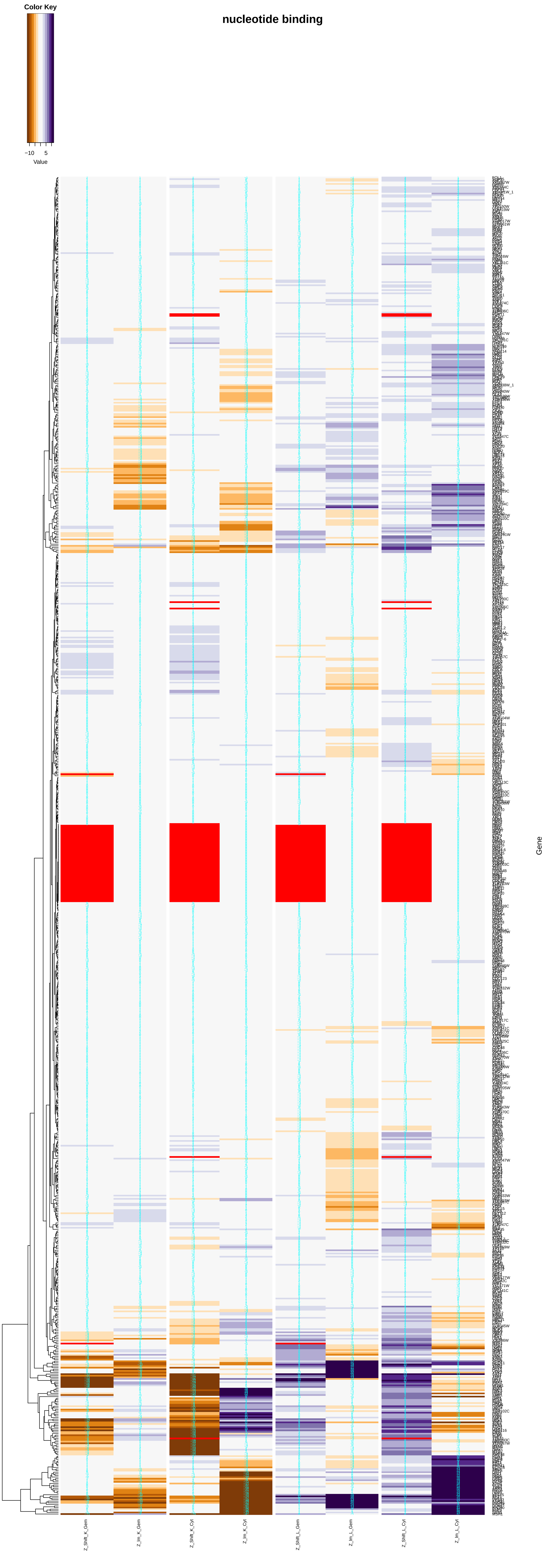
nucleotide binding
Color Key
−10
5
Value
Gene
Z_Shift_K_Gem
Z_Im_K_Gem
Z_Shift_K_Cyt
Z_Im_K_Cyt
Z_Shift_L_Gem
Z_Im_L_Gem
Z_Shift_L_Cyt
Z_Im_L_Cyt

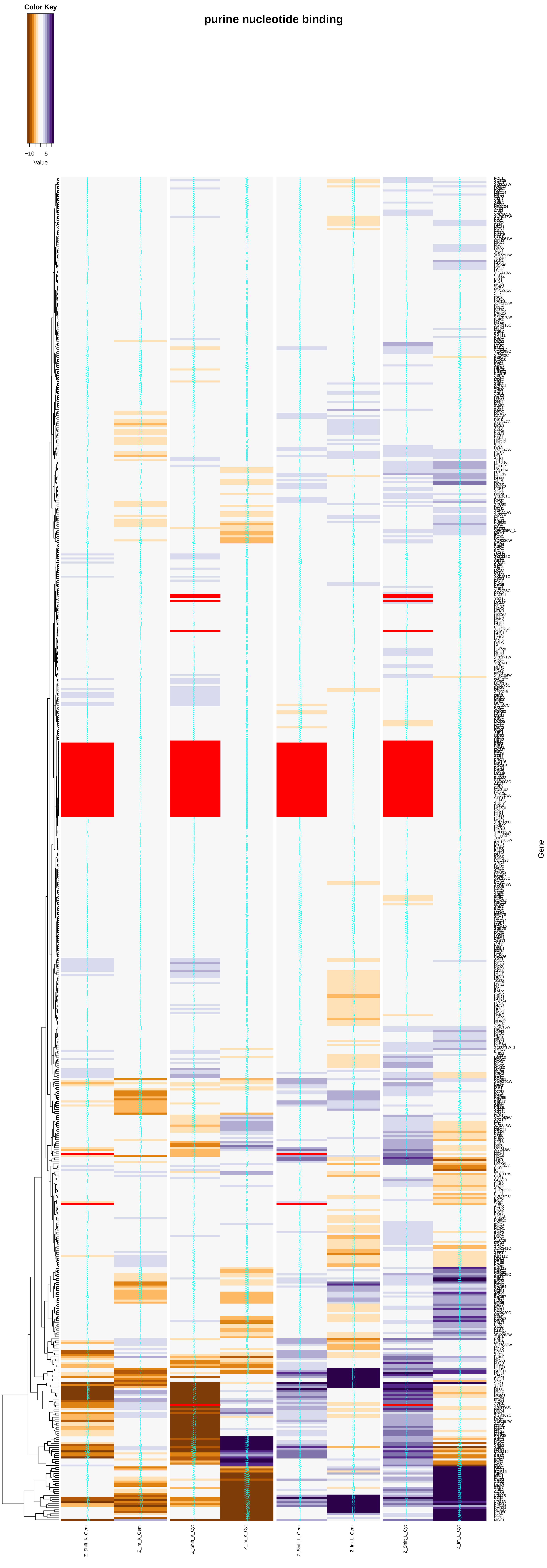

purine nucleotide binding
Color Key
−10
5
Value
Gene
Z_Shift_K_Gem
Z_Im_K_Gem
Z_Shift_K_Cyt
Z_Im_K_Cyt
Z_Shift_L_Gem
Z_Im_L_Gem
Z_Shift_L_Cyt
Z_Im_L_Cyt

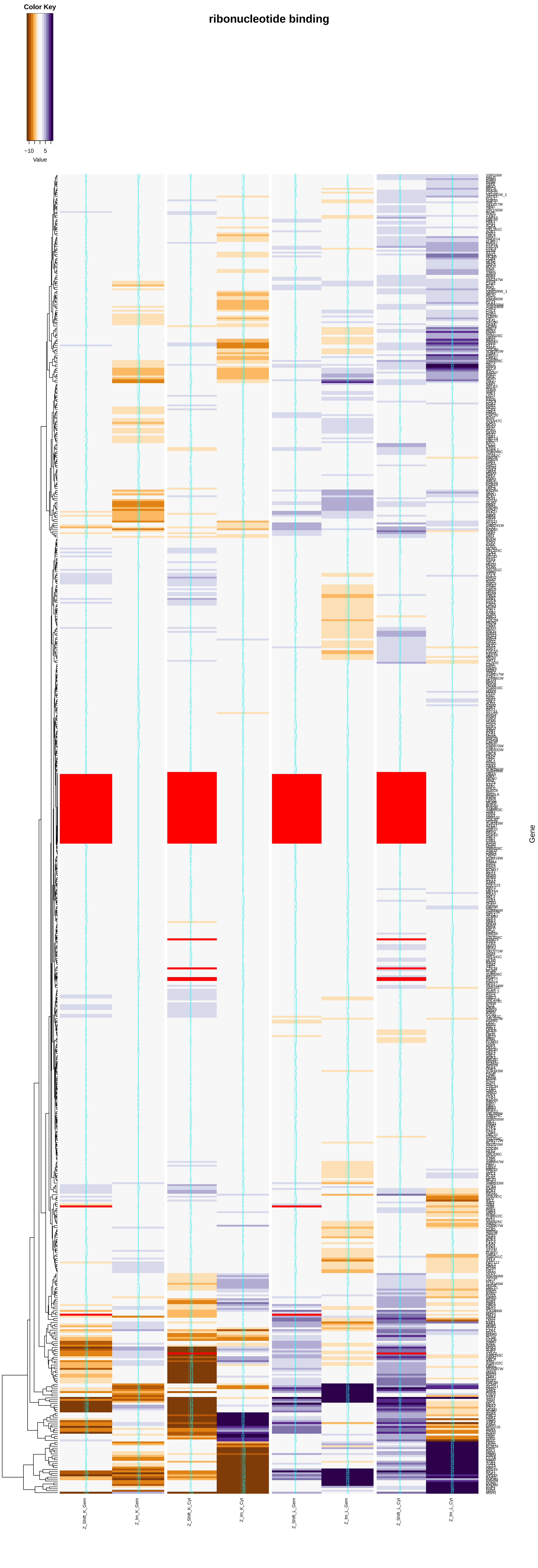
ribonucleotide binding
Color Key
−10
5
Value
Z_Shift_K_Gem
Z_Im_K_Gem
Z_Shift_K_Cyt
Z_Im_K_Cyt
Z_Shift_L_Gem
Z_Im_L_Gem
Z_Shift_L_Cyt
Z_Im_L_Cyt
Gene

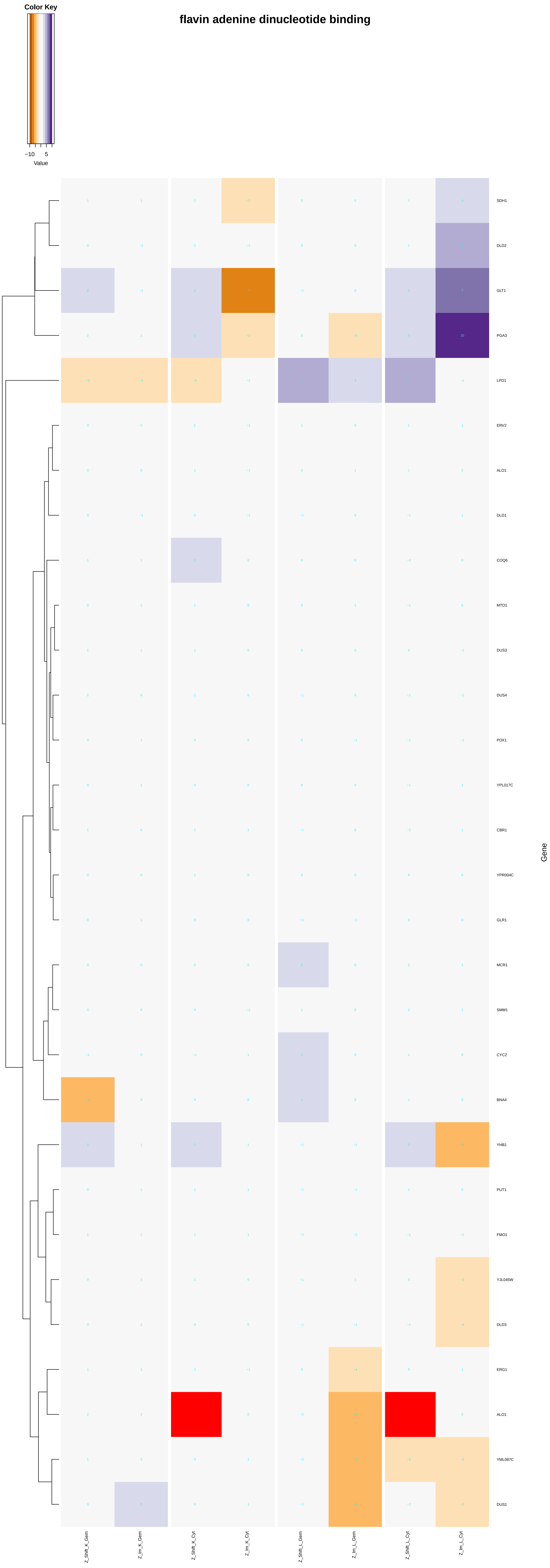
flavin adenine dinucleotide binding
Color Key
−10
5
Value
SDH1
DLD2
GLT1
PGA3
LPD1
ERV2
ALO1
DLD1
COQ6
MTO1
DUS3
DUS4
POX1
YPL017C
CBR1
YPR004C
GLR1
MCR1
SMM1
CYC2
BNA4
YHB1
PUT1
FMO1
YJL045W
DLD3
ERG1
ALO1
YML087C
DUS1
Gene
Z_Shift_K_Gem
Z_Im_K_Gem
Z_Shift_K_Cyt
Z_Im_K_Cyt
Z_Shift_L_Gem
Z_Im_L_Gem
Z_Shift_L_Cyt
Z_Im_L_Cyt

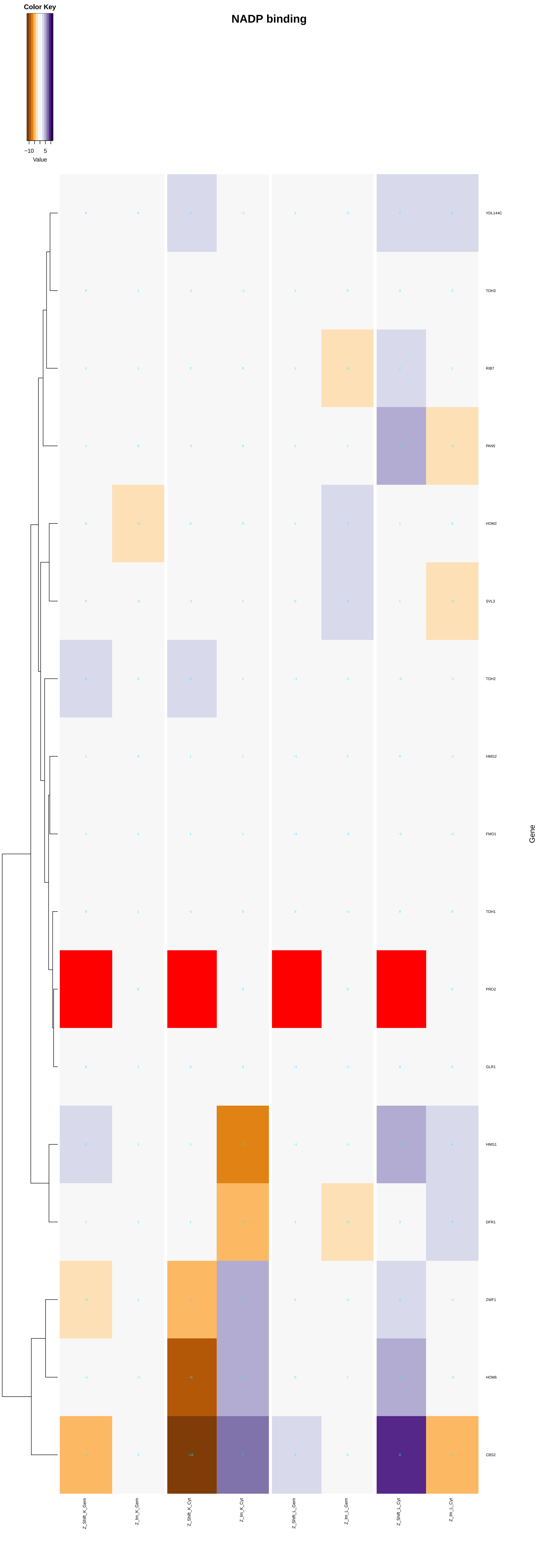

Color Key
−10
5
Value
NADP binding
YDL144C
TDH3
RIB7
PAN5
HOM2
SVL3
TDH2
HMG2
FMO1
TDH1
PRO2
GLR1
HMG1
DFR1
ZWF1
HOM6
CBS2
Gene
Z_Shift_K_Gem
Z_Im_K_Gem
Z_Shift_K_Cyt
Z_Im_K_Cyt
Z_Shift_L_Gem
Z_Im_L_Gem
Z_Shift_L_Cyt
Z_Im_L_Cyt

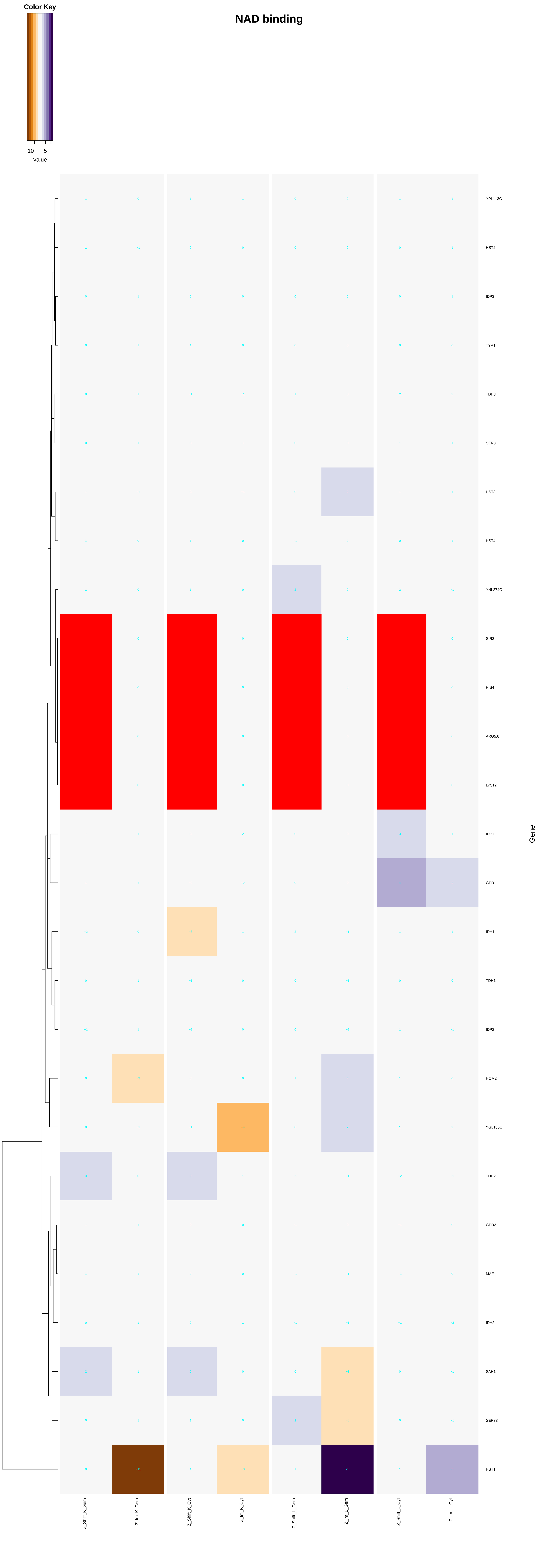

Color Key
−10
5
Value
NAD binding
YPL113C
HST2
IDP3
TYR1
TDH3
SER3
HST3
HST4
YNL274C
SIR2
HIS4
ARG5,6
LYS12
IDP1
GPD1
IDH1
TDH1
IDP2
HOM2
YGL185C
TDH2
GPD2
MAE1
IDH2
SAH1
SER33
HST1
Gene
Z_Shift_K_Gem
Z_lm_K_Gem
Z_Shift_K_Cyt
Z_lm_K_Cyt
Z_Shift_L_Gem
Z_lm_L_Gem
Z_Shift_L_Cyt
Z_lm_L_Cyt

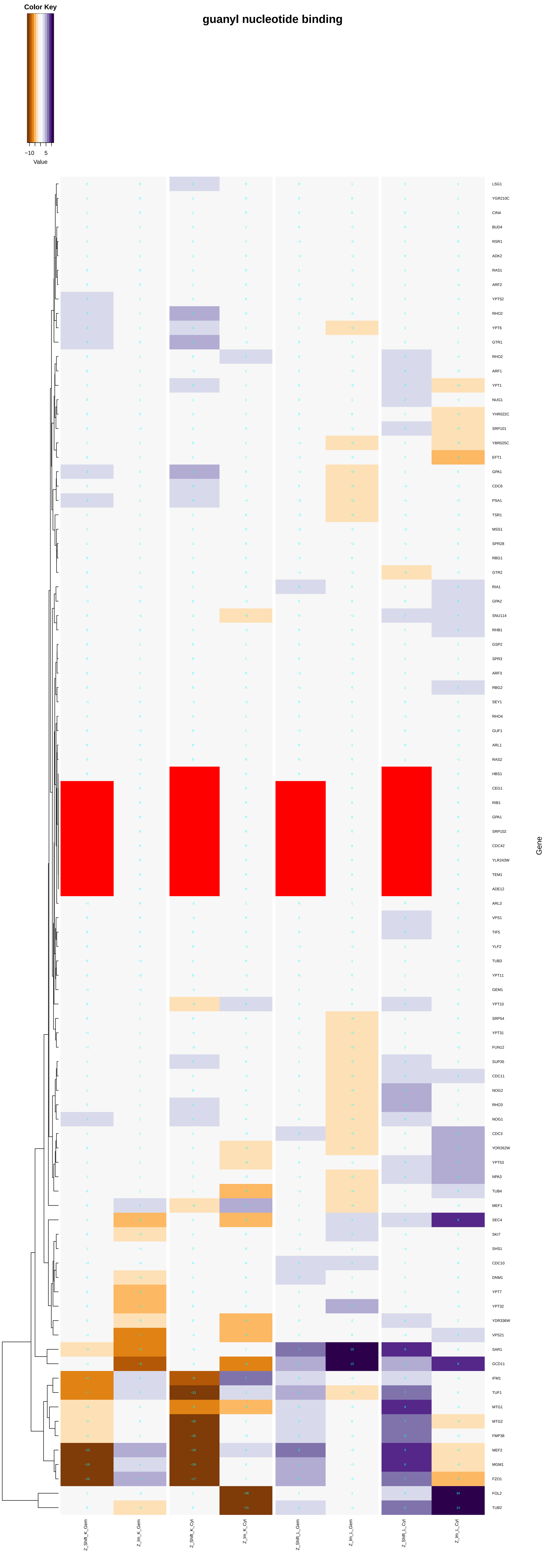

guanyl nucleotide binding
Color Key
−10
5
Value
Gene
LSG1
YGR210C
CIN4
BUD4
RSR1
ADK2
RAS1
ARF2
YPT52
RHO2
YPT6
GTR1
RHO2
ARF1
YPT1
NUG1
YHR022C
SRP101
YBR025C
EFT1
GPA1
CDC6
PSA1
TSR1
MSS1
SPR28
RBG1
GTR2
RIA1
GPA2
SNU114
RHB1
GSP2
SPR3
ARF3
RBG2
SEY1
RHO4
GUF1
ARL1
RAS2
HBS1
CEG1
RIB1
GPA1
SRP102
CDC42
YLR243W
TEM1
ADE12
ARL3
VPS1
TIF5
YLF2
TUB3
YPT11
GEM1
YPT10
SRP54
YPT31
FUN12
SUP35
CDC11
NOG2
RHO3
NOG1
CDC3
YOR262W
YPT53
NPA3
TUB4
MEF1
SEC4
SKI7
SHS1
CDC10
DNM1
YPT7
YPT32
YDR336W
VPS21
SAR1
GCD11
IFM1
TUF1
MTG1
MTG2
FMP38
MEF2
MGM1
FZO1
FOL2
TUB2
Z_Shift_K_Gem
Z_Im_K_Gem
Z_Shift_K_Cyt
Z_Im_K_Cyt
Z_Shift_L_Gem
Z_Im_L_Gem
Z_Shift_L_Cyt
Z_Im_L_Cyt

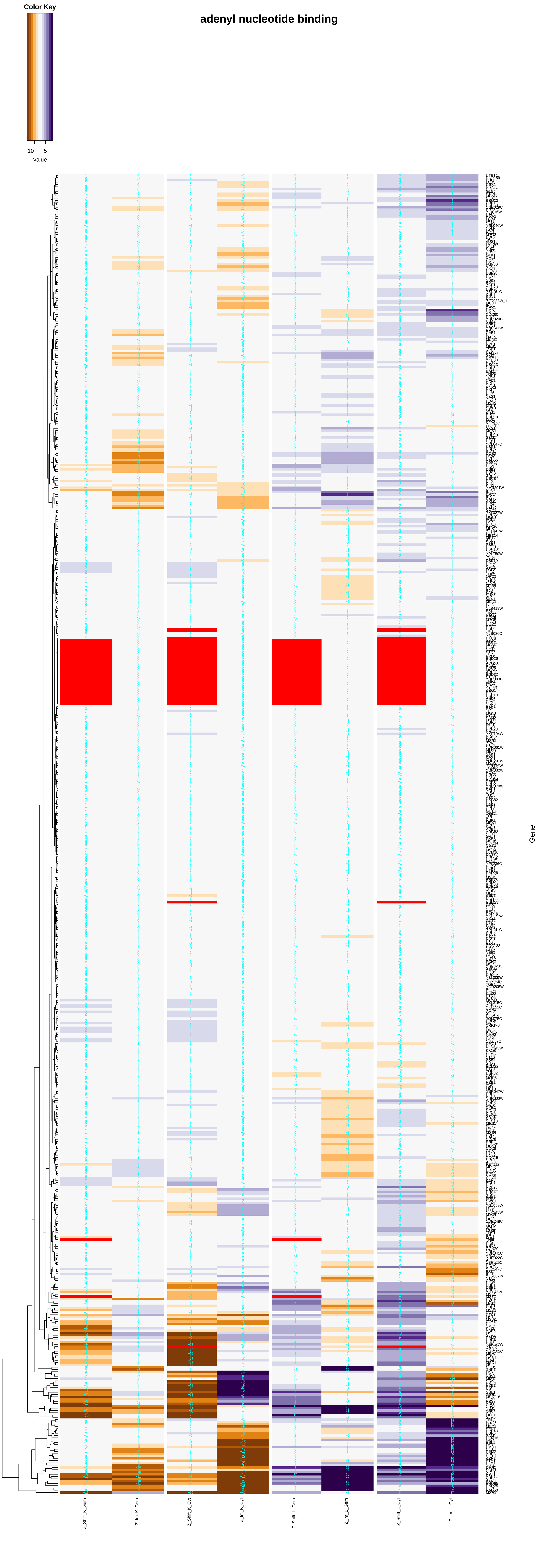
adenyl nucleotide binding
Color Key
−10
5
Value
Gene
Z_Shift_K_Gem
Z_Im_K_Gem
Z_Shift_K_Cyt
Z_Im_K_Cyt
Z_Shift_L_Gem
Z_Im_L_Gem
Z_Shift_L_Cyt
Z_Im_L_Cyt

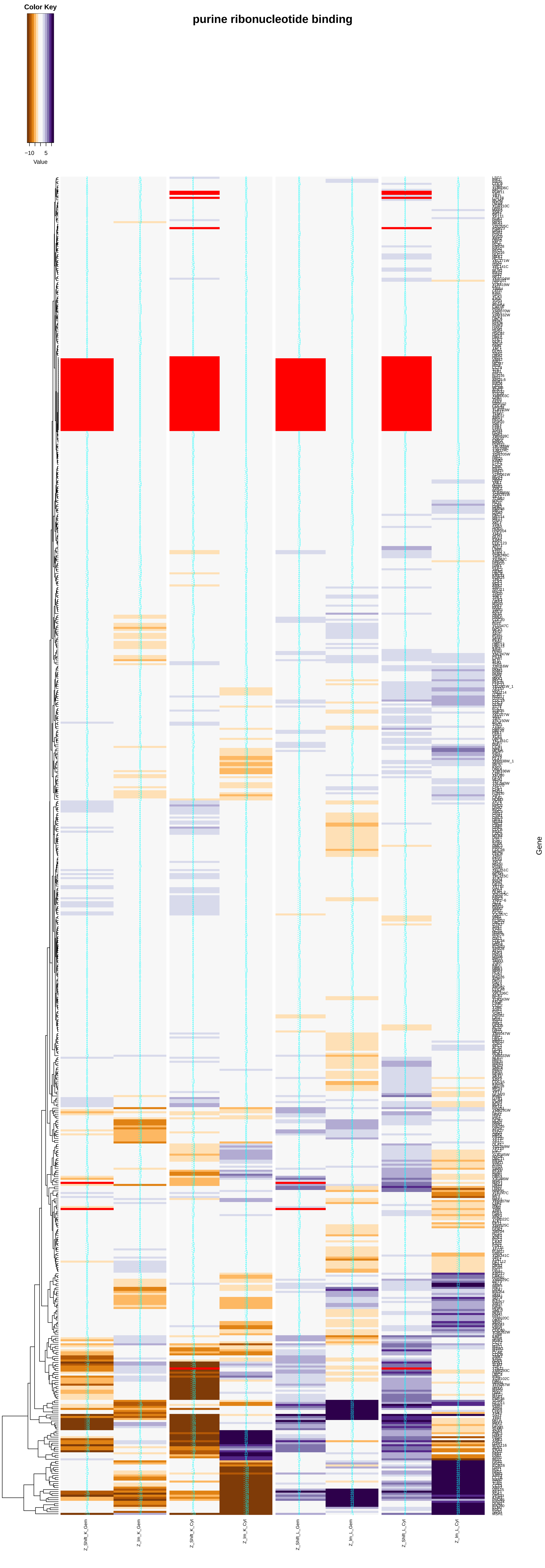
Color Key
purine ribonucleotide binding
−10
5
Value
Z_Shift_K_Gem
Z_Im_K_Gem
Z_Shift_K_Cyt
Z_Im_K_Cyt
Z_Shift_L_Gem
Z_Im_L_Gem
Z_Shift_L_Cyt
Z_Im_L_Cyt
Gene

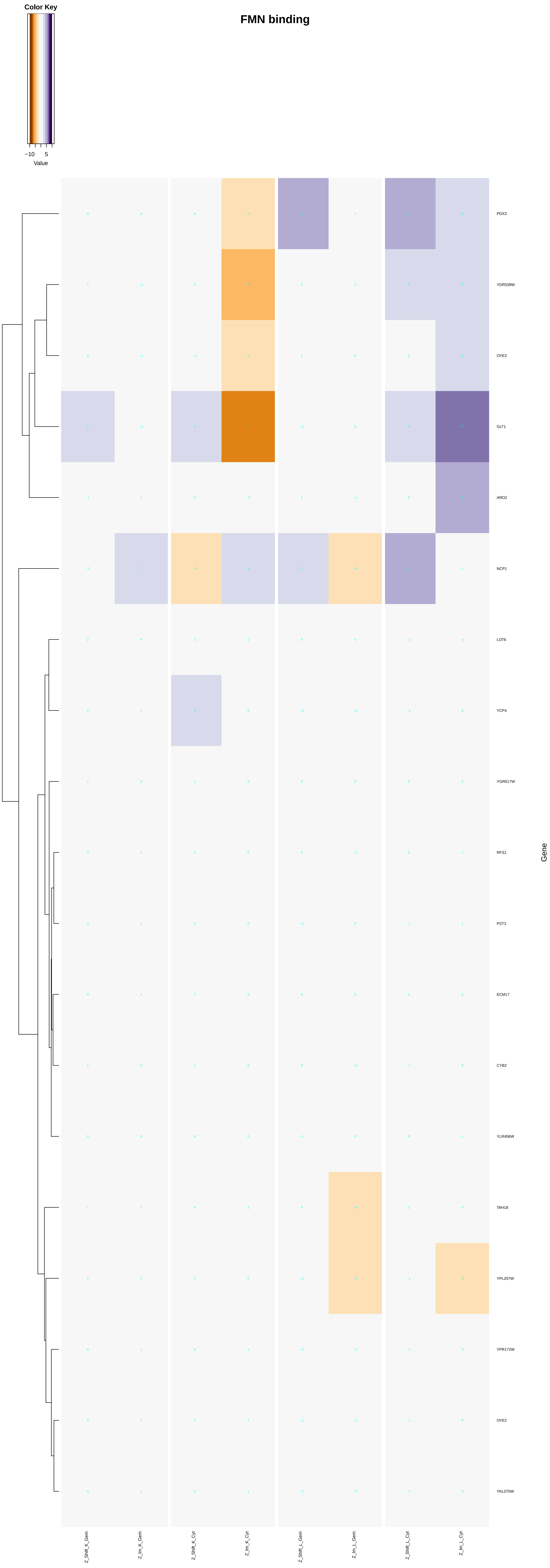

FMN binding
Color Key
−10
5
Value
PDX3
YDR539W
OYE3
GLT1
ARO2
NCP1
LOT6
YCP4
YGR017W
RFS1
PST2
ECM17
CYB2
YLR456W
TAH18
YPL207W
YPR172W
OYE2
YKL070W
Gene
Z_Shift_K_Gem
Z_Im_K_Gem
Z_Shift_K_Cyt
Z_Im_K_Cyt
Z_Shift_L_Gem
Z_Im_L_Gem
Z_Shift_L_Cyt
Z_Im_L_Cyt

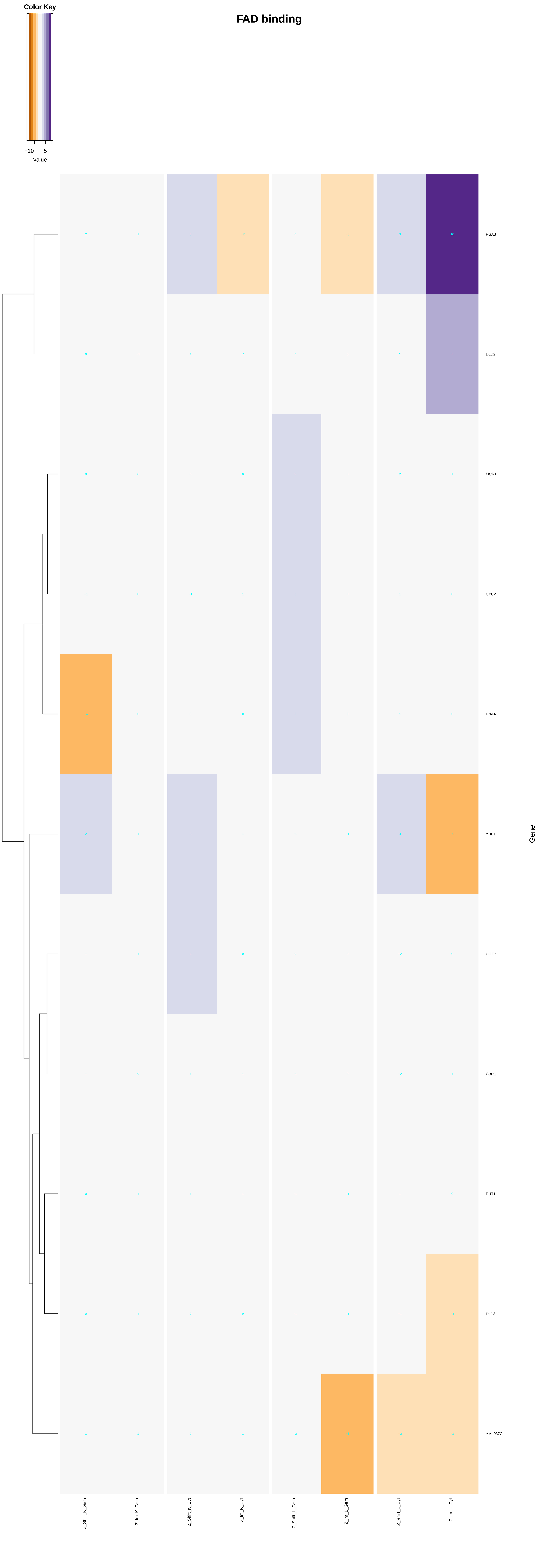
FAD binding
Color Key
−10
5
Value
PGA3
DLD2
MCR1
CYC2
BNA4
YHB1
COQ6
CBR1
PUT1
DLD3
YML087C
Gene
Z_Shift_K_Gem
Z_Im_K_Gem
Z_Shift_K_Cyt
Z_Im_K_Cyt
Z_Shift_L_Gem
Z_Im_L_Gem
Z_Shift_L_Cyt
Z_Im_L_Cyt

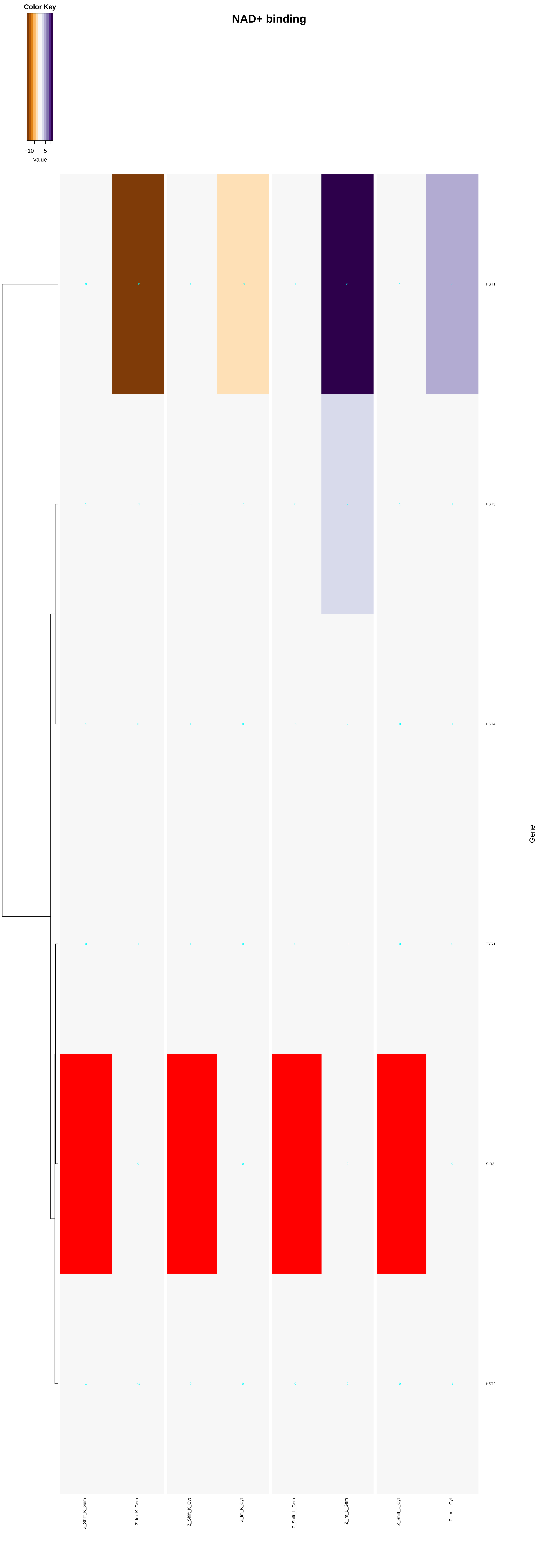

NAD+ binding
Color Key
−10
5
Value
HST1
HST3
HST4
TYR1
SIR2
HST2
Gene
Z_Shift_K_Gem
Z_Im_K_Gem
Z_Shift_K_Cyt
Z_Im_K_Cyt
Z_Shift_L_Gem
Z_Im_L_Gem
Z_Shift_L_Cyt
Z_Im_L_Cyt

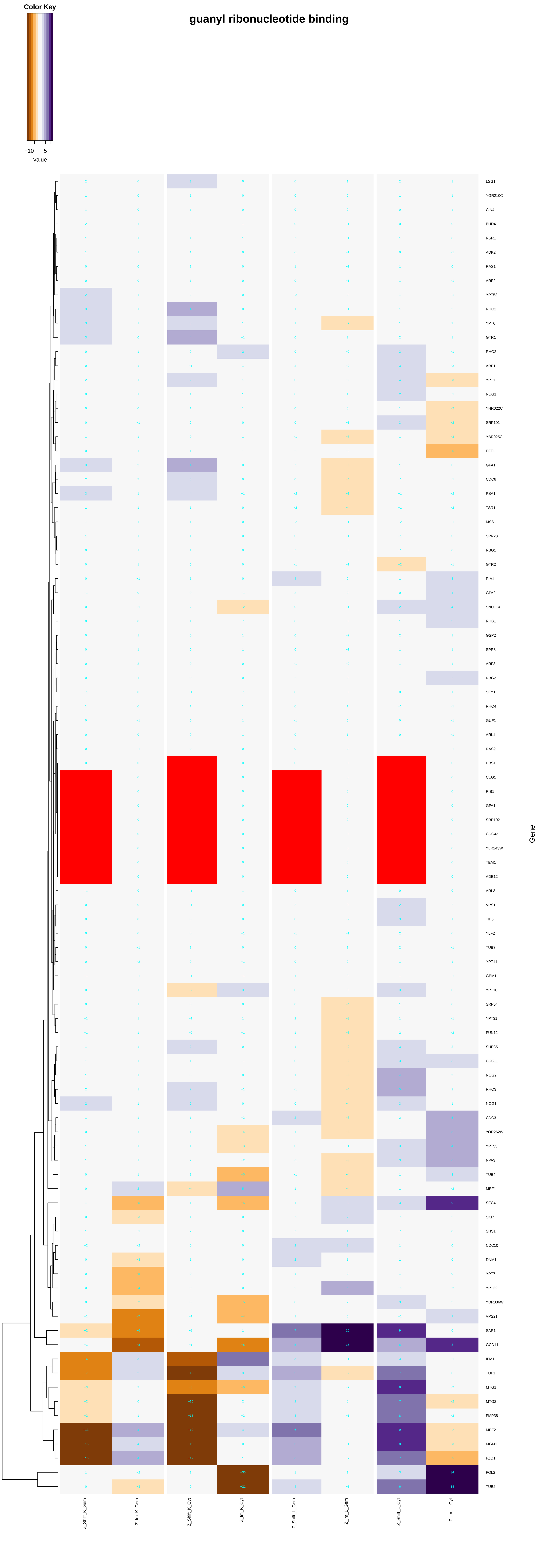

guanyl ribonucleotide binding
Color Key
−10
5
Value
LSG1
YGR210C
CIN4
BUD4
RSR1
ADK2
RAS1
ARF2
YPT52
RHO2
YPT6
GTR1
RHO2
ARF1
YPT1
NUG1
YHR022C
SRP101
YBR025C
EFT1
GPA1
CDC6
PSA1
TSR1
MSS1
SPR28
RBG1
GTR2
RIA1
GPA2
SNU114
RHB1
GSP2
SPR3
ARF3
RBG2
SEY1
RHO4
GUF1
ARL1
RAS2
HBS1
CEG1
RIB1
GPA1
SRP102
CDC42
YLR243W
TEM1
ADE12
ARL3
VPS1
TIF5
YLF2
TUB3
YPT11
GEM1
YPT10
SRP54
YPT31
FUN12
SUP35
CDC11
NOG2
RHO3
NOG1
CDC3
YOR262W
YPT53
NPA3
TUB4
MEF1
SEC4
SKI7
SHS1
CDC10
DNM1
YPT7
YPT32
YDR336W
VPS21
SAR1
GCD11
IFM1
TUF1
MTG1
MTG2
FMP38
MEF2
MGM1
FZO1
FOL2
TUB2
Gene
Z_Shift_K_Gem
Z_Im_K_Gem
Z_Shift_K_Cyt
Z_Im_K_Cyt
Z_Shift_L_Gem
Z_Im_L_Gem
Z_Shift_L_Cyt
Z_Im_L_Cyt

adenyl−nucleotide exchange factor activity

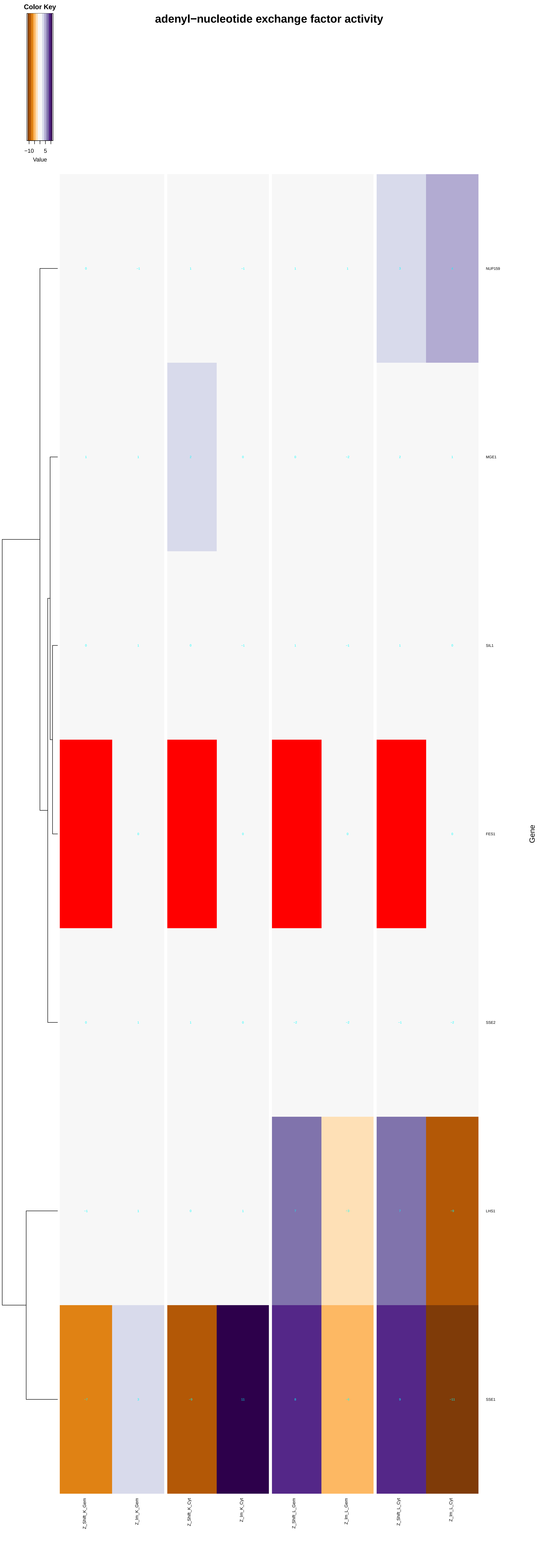

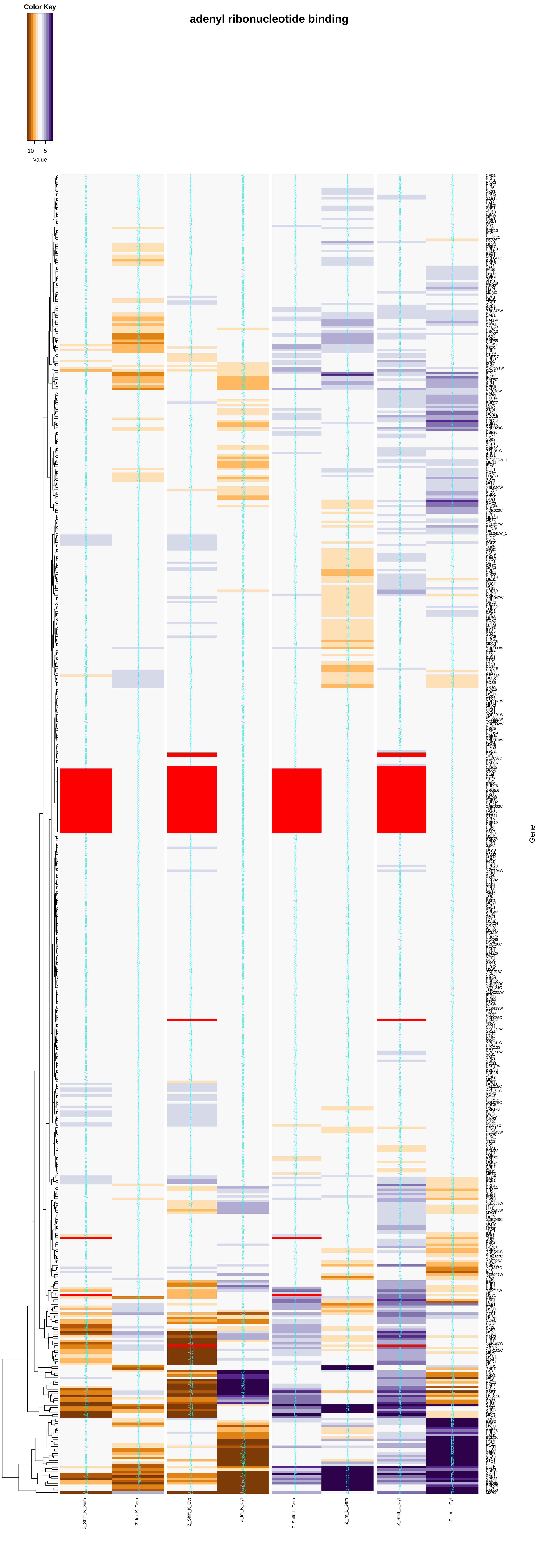
Color Key
−10
5
Value
adenyl ribonucleotide binding
Gene
Z_Shift_K_Gem
Z_Im_K_Gem
Z_Shift_K_Cyt
Z_Im_K_Cyt
Z_Shift_L_Gem
Z_Im_L_Gem
Z_Shift_L_Cyt
Z_Im_L_Cyt

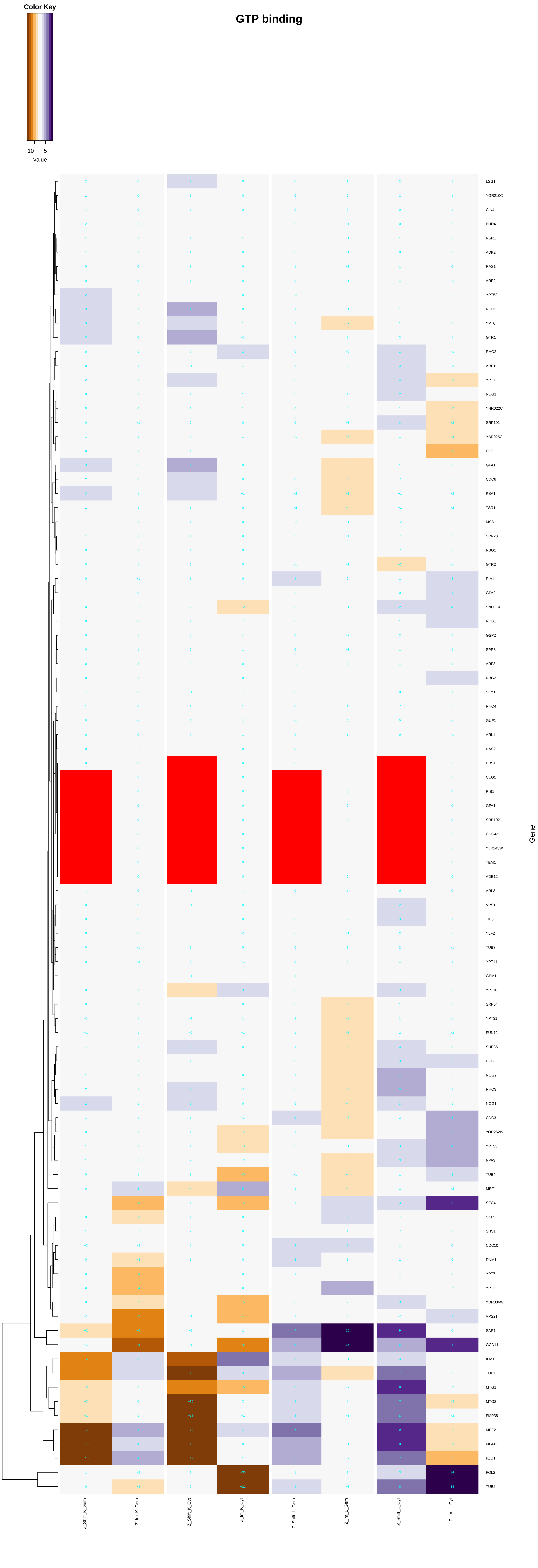
GTP binding
Color Key
−10
5
Value
LSG1
YGR210C
CIN4
BUD4
RSR1
ADK2
RAS1
ARF2
YPT52
RHO2
YPT6
GTR1
RHO2
ARF1
YPT1
NUG1
YHR022C
SRP101
YBR025C
EFT1
GPA1
CDC6
PSA1
TSR1
MSS1
SPR28
RBG1
GTR2
RIA1
GPA2
SNU114
RHB1
GSP2
SPR3
ARF3
RBG2
SEY1
RHO4
GUF1
ARL1
RAS2
HBS1
CEG1
RIB1
GPA1
SRP102
CDC42
YLR243W
TEM1
ADE12
ARL3
VPS1
TIF5
YLF2
TUB3
YPT11
GEM1
YPT10
SRP54
YPT31
FUN12
SUP35
CDC11
NOG2
RHO3
NOG1
CDC3
YOR262W
YPT53
NPA3
TUB4
MEF1
SEC4
SKI7
SHS1
CDC10
DNM1
YPT7
YPT32
YDR336W
VPS21
SAR1
GCD11
IFM1
TUF1
MTG1
MTG2
FMP38
MEF2
MGM1
FZO1
FOL2
TUB2
Gene
Z_Shift_K_Gem
Z_Im_K_Gem
Z_Shift_K_Cyt
Z_Im_K_Cyt
Z_Shift_L_Gem
Z_Im_L_Gem
Z_Shift_L_Cyt
Z_Im_L_Cyt

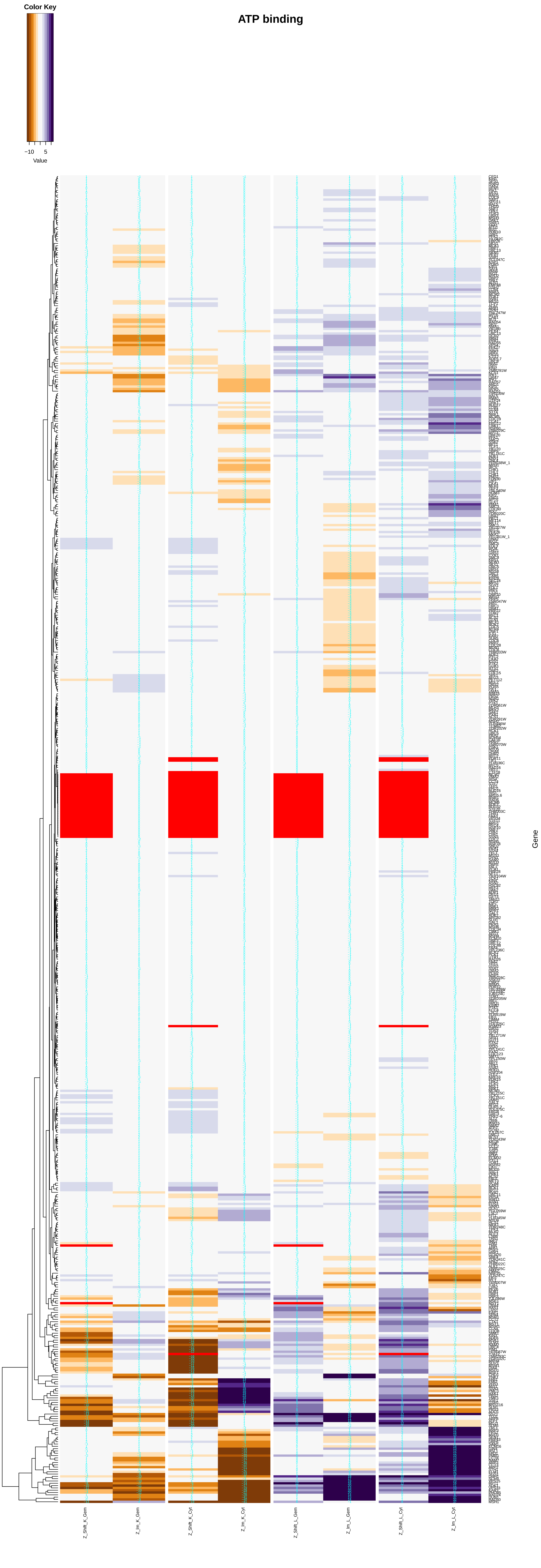
ATP binding
Color Key
−10
5
Value
Z_Shift_K_Gem
Z_Im_K_Gem
Z_Shift_K_Cyt
Z_Im_K_Cyt
Z_Shift_L_Gem
Z_Im_L_Gem
Z_Shift_L_Cyt
Z_Im_L_Cyt
Gene

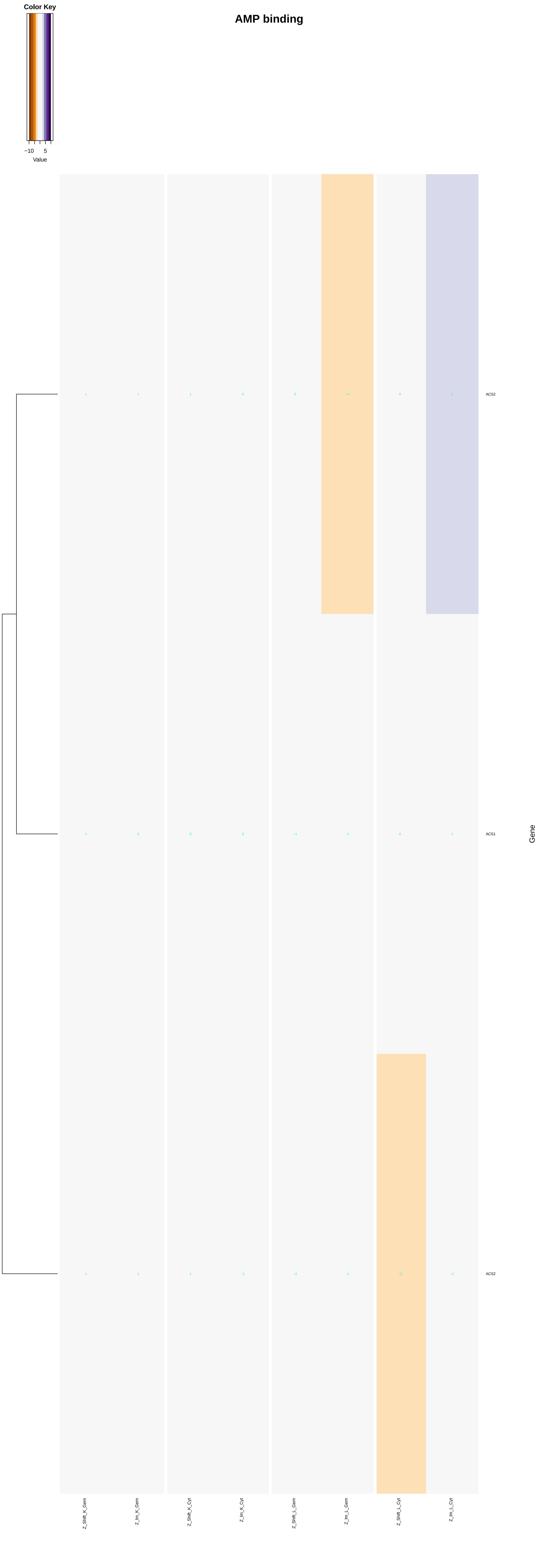
Color Key
−10
5
Value
AMP binding
ACS2
ACS1
ACS2
Gene
Z_Shift_K_Gem
Z_Im_K_Gem
Z_Shift_K_Cyt
Z_Im_K_Cyt
Z_Shift_L_Gem
Z_Im_L_Gem
Z_Shift_L_Cyt
Z_Im_L_Cyt
